# Post-stroke dysphagia: An exploration of initial identification and management performed by nurses and doctors

**DOI:** 10.4102/sajcd.v67i1.625

**Published:** 2020-05-28

**Authors:** Maggie Pierpoint, Mershen Pillay

**Affiliations:** 1 Discipline of Speech-Language Pathology, School of Health Sciences, University of KwaZulu-Natal, Durban, South Africa; 2 Discipline of Speech-Language Pathology, School of Health Sciences, University of KwaZulu-Natal, Durban, South Africa

**Keywords:** stroke, dysphagia management, early identification, dysphagia intervention, doctors and registered nurses

## Abstract

**Background:**

South African speech-language therapists are the only health professionals charged with dysphagia rehabilitation. However, registered nurses and doctors are often initial healthcare contact points for post-stroke dysphagia. Notwithstanding service concerns, they do indeed identify and manage post-stroke dysphagia. However, little is known about specifically what they do during these initial clinical encounters.

**Objective:**

To explore how doctors and registered nurses, on initial clinical contact, identify and manage post-stroke dysphagia.

**Method:**

A quantitative descriptive survey design, with non-probability, purposive sampling, was used. Twenty-one registered nurses and four doctors at a private South African hospital self-administered a questionnaire. Univariate analyses were completed by calculating frequency distributions of nurses’ and doctors’ identification and management practices.

**Results:**

Most (86%) did not use a formal screening tool. Indicators screened informally included: presence of drooling (84%) or gag reflex (76%), level of alertness (80%) and spontaneous saliva swallow (80%). Participants neglected important indicators like voluntary cough and vocal quality. Management provided included head of bed elevation (96%), speech-language therapist referrals (92%), nasogastric tube insertions (88%), intravenous fluids (84%) and positional adjustments (76%). Alternative management included total parenteral nutrition (52%), syringe feeding (48%), swallow muscle strengthening exercises (56%) and swallow manoeuvres (52%).

**Conclusion:**

Results indicated that doctors and registered nurses under-utilised important dysphagia indicators and used potentially harmful management practices like syringe feeding. Management practices further included out-of-scope methods like dysphagia rehabilitation exercises or manoeuvres. Recommendations include peer dysphagia screening training using formal tools and basic dysphagia management methods to better equip doctors and registered nurses when they clinically engage post-stroke patients.

## Introduction

Globally ~590 million people (8% of the world population) experience dysphagia (Cichero et al., 2017). In South Africa (SA), Blackwell and Littlejohns (2010) reported a prevalence of dysphagia in 56% of stroke cases, but indicated that this number may have been exaggerated because of the use of subjective and conservative swallowing assessment methods. Patients with dysphagia (post-stroke) are more at risk of developing pneumonia (22.9%), which is most likely aspiration-related than those without dysphagia (1.1%). Therefore, they have an increased risk of mortality, and early identification and management is essential to manage aspiration risks (Arnold et al., 2016).

It is important to disclaim that we refer to a bespoke South African reality about dysphagia practice in SA as reviewed by Andrews and Pillay (2017). They referred to a practice reality that, prior to a Speech-Language Therapist (SLT) assessment or diagnosis, it is highly likely that post-stroke dysphagics will be identified and managed by other healthcare professionals, usually nurses and medical doctors. In this light, the terms ‘identification’ and ‘management’ take on the uncommon usage of referring to what occurs before the more specialised SLT-led screening and management.

Identification (or screening) of dysphagia refers to that which occurs through formal or informal methods (American Speech-Language-Hearing Association [ASHA], 2009; Donovan et al., 2013; Hinchey et al., 2005). Informal screening refers to focussed observation of clinical signs and symptoms and generally does not include the use of a formal screening tool (ASHA, 2009; Bryer et al., 2010). Formal screening refers to the use of a systematic method (e.g. checklist) with specified pass or fail criteria (Donovan et al., 2013) to identify the presence or absence of dysphagia requiring further in-depth assessment by an SLT (Bray et al., 2016; Horiguchi & Suzuki, 2011).

Management of dysphagia, usually within an SLTs’ scope of practice, may include diet textural modifications, compensatory postures and therapeutic exercises to name but a few methods to improve the safety of the swallow (Rogus-Pulia & Robbins, 2013). Intervention is that which is performed to maintain or improve the patient’s current health status and to prevent complications associated with the patient’s dysphagia (Groher & Crary, 2010).

The incidence of pneumonia may increase by 1% per day when identification is delayed (Bray et al., 2016). South African Stroke Guidelines recommended that all stroke survivors should have a swallowing assessment before oral intake. To guide this process, a list of items is provided to assist an observational (informal) screening with recommendation that if these signs are present, then the patient should remain nil per mouth until formally assessed by an SLT. It is not clearly indicated who should perform the initial screening (Bryer et al., 2010). Perhaps, this is because doctors and registered nurses are the first point of contact (Andrews & Pillay, 2017). These professionals then refer to SLTs for dysphagia assessment, differential diagnosis and treatment (ASHA, 2015; Blackwell & Littlejohns, 2010).

In SA, there are insufficient SLTs to manage the demand (Pillay, Tiwari, Kathard, & Chikte, in press). While SLTs manage dysphagia, only a small percentage actually provide comprehensive dysphagia services in SA, as evidenced by the number of respondents in a dysphagia practice survey (Andrew & Pillay, 2017). The implication of this workforce shortage is that persons with post-stroke dysphagia may experience unnecessary disruptions in nutrition, hydration and medication that can adversely affect their general health (Blackwell & Littlejohns, 2010; Bryer et al., 2010; Donovan et al., 2013). To bridge the gap between the shortage of SLTs in SA and the need for timely dysphagia identification and intervention, task shifting and role sharing is recommended (World Health Organization [WHO], 2008) especially for tasks that require basic training such as screening or identification.

Indeed, several countries (e.g. the United Kingdom, the United States and Canada) have programme for swallowing screening to be conducted by other trained professionals with the use of a validated screening tool, for example, The Modified Mann Assessment of Swallowing Ability (MMASA) (for doctors) or the Toronto Bedside Swallowing Screening (TOR-BSST) for doctors and registered nurses (Antonios et al., 2010; Hebert et al., 2016; Intercollegiate Stroke Working Party, 2016; Martino et al., 2009; Winstein et al., 2016). Doctors and registered nurses may be trained to perform the initial swallowing screening, as they are the first healthcare practitioners that post-stroke patients encounter and are more readily available than SLTs (Antonios et al., 2010; Pillay & Kathard, 2018; Titsworth et al., 2013). This not only has the potential to reduce the time to screening but can also reduce pneumonia rates and hospital length of stay, especially when coupled with appropriate early intervention (Palli et al., 2017).

Alongside most health workers in SA, 78% SLTs work in the private sector (Pillay et al, in press), continuing an established tradition of over-servicing a minority of South Africans who can afford and access their services (Pillay & Kathard, 2018). In the section on methodology, the study site is described and a motivation provided for why it was selected. Suffice to note here that this private sector was deemed an interesting, fertile context to study how nurses and doctors who theoretically should be more aware of, and have access to, SLT services practise with post-stroke dysphagia. Specifically, we aimed to describe how doctors and registered nurses: (1) identify and (2) manage persons with post-stroke dysphagia.

## Research methods and design

### Design

A cross-sectional, descriptive survey (Babbie & Mouton, 2014; eds. Franklin & Walker, 2010) was performed at a single, private acute care hospital in SA to gather first-hand information in a timely and cost-effective manner about the dysphagia practices of doctors and registered nurses. The study location was a private hospital because it is purposively aligned to the study question, reflected the dominant practice site for SLT in SA (notwithstanding underservicing in the public sector) and was a convenient practice work site for the primary researcher. This article is part of a larger study conducted for a master’s degree and only the main objectives and results are discussed here. The reader is referred to Pierpoint (2016).

### Data collection method and tool

A paper-based questionnaire (Appendix 1) was compiled by the first author and self-administered. The questionnaire was in English – the hospital’s official language of business.

### Participants

The study population, selected by non-probability purposive sampling, consisted of doctors and registered nurses (74) who practice within this hospital. Doctors were included based on their specialty (those who are typically involved in stroke cases) and registered nurses based on the wards in which stroke patients are treated. They consisted of 11 medical doctors (three physicians, three specialist physicians, one neurologist and four neurosurgeons) and 63 registered nurses (23 from the intensive care unit, 18 from high care and 22 from the cardiothoracic and medical wards). Twenty-one registered nurses and four doctors agreed to complete the DMQ, resulting in a response rate of 33.3% for the former and 36.4% for the latter. The overall study response rate was 33.78%.

### Pilot study

The DMQ was developed for this study, which necessitated a pilot study. The DMQ was based on dysphagia risk factors, signs, symptoms, screening and intervention and derived from studies by Blackwell and Littlejohns (2010), Baroni, Fábio and Dantas (2012), Carnaby, Hankey and Pizzi (2006), Horiguchi and Suzuki (2011), Rogus-Pulia and Robbins (2013), Smith Hammond and Goldstein (2006) as well as Via and Mechanick (2013). An overview of the questionnaire is presented in Table 1 according to the study objectives. The aim of this pilot study was to pre-test the questionnaire to determine whether the questions and instructions were stated clearly enough and were understood correctly by the participants. The DMQ included items specific to the scope of practice of dysphagia practitioners like SLTs. It is recognised that these rehabilitation methods, for example, airway protection manoeuvres, require training and competence and were included to establish this very fact as well as to provide a wide as possible map of practice activities. Notably, these items, including the dysphagia rehabilitation items, were reviewed and are the product of the pilot study.

**TABLE 1:** Objectives and methods of development of the Dysphagia Management Questionnaire.

Number	Objectives	Types of questions	Questions
1	To describe how doctors and registered nurses identify dysphagia.	A two-choice question was used to enquire about the use of a formal screening test, with a section for specifying which test was used (question 1.2)	1.2 Do you use a specific screening test or checklist to assess the presence or absence of dysphagia? (choose one answer)
		One checklist question with 14 options and an ‘other’ section was used to determine what doctors and registered nurses screen for (formally or informally) (question 1.3)	1.3 What do you assess or observe (formally or informally) to determine whether or not the patient has dysphagia? (Please tick all the relevant boxes)
2	To describe how doctors and registered nurses manage dysphagia once it has been identified.	Two checklist questions (question 2.1 and 2.2) were used.	2.1 What do you do once you have identified that a patient has dysphagia? (Please tick all the relevant boxes)
		The first question (question 2.1) enquired about the use of enteral and parenteral feeding, compensations, rehabilitation and referral. The question had five subcategories with 24 options in total and an ‘other’ section in each subcategory.	2.2 When a patient is tube fed (e.g. percutaneous endoscopic gastroscopy [PEG] or nasogastric [NG]) because of a swallowing difficulty, how do you decide if they can eat orally again? (Please tick all the relevant boxes)
		The second question (question 2.2) enquired about the intervention of persons who are tube fed, in terms of returning to oral feeding. This question consisted of four options that could be marked.	-

Pilot study participants included three doctors and six registered nurses from a different private subacute care rehabilitation hospital who matched the target, main study population.

A pre-testing technique, namely, concurrent cognitive interviews, was used for the pilot study (Caspar & Peytcheva, 2011). This technique required the participants to individually complete the DMQ and comment on the questions or ask for clarification in case of ambiguity (Caspar & Peytcheva, 2011). The results and suggestions were used to alter the questionnaire, to ensure optimal validity and reliability of the research instrument (Babbie & Mouton, 2014).

### Data collection process

The questionnaires were delivered to the relevant doctors and registered nurses, followed by a postal reminder of the collection date. Completed consent forms and questionnaires were collected and sorted by an independent entity to ensure anonymity.

### Data analysis and management

After data collection and editing, a custom-designed data import utility, with a user interface identical to the DMQ, was used to capture the raw data and convert it into a Microsoft Excel spreadsheet with numerical data. Data validation was coded into the data import utility to prevent user error when capturing the data. Univariate analysis of the numerical data was performed to describe the single variables in the study objectives: (1) identification of dysphagia and (2) management of dysphagia. The numerical data were analysed using descriptive statistics, and frequency distributions were calculated by the statistician with the use of Microsoft Excel and presented in tables for the interpretation of the results.

### Ethical consideration

Ethical approval was obtained from the University of Kwazulu-Natal’s Biomedical Research Ethics Committee (BE280/15). Gatekeeper permission was obtained from the hospital management, and participants were asked to sign an informed consent document before completing the Dysphagia Management Questionnaire (DMQ), which was designed for this study.

## Results

The results are described according to how doctors and registered nurses: (1) identify and (2) manage dysphagia. All 25 participants were experienced in working with stroke patients, most (72%) indicated that they had experience working in the intensive care unit, their years of experience ranged from five to 20+ years, and 96% indicated that they were experienced in identifying dysphagia.

### Dysphagia identification

Seventy-five per cent (3 of 4) of the doctors and 89% (16 of 18) of the registered nurses identified dysphagia through informal screening methods. See Table 2 for a full list of the items screened, which included the presence of drooling, level of alertness and ability to swallow their saliva reflexively. The ‘other’ items that participants reported were ‘Bell’s palsy’ and ‘turning the head to the side when swallowing’.

**TABLE 2:** Items screened to determine the presence of dysphagia.

Number	Items	Doctors (*n* = 4)		Registered nurses (*n* = 21)		Total (*n* = 25)	
		Number	%	Number	%	Number	%
1	Presence of drooling	3	75	17	81	20	80
2	Level of alertness	4	100	16	76	20	80
3	Ability to swallow the saliva reflexively or spontaneously	4	100	16	76	20	80
4	Presence of a gag reflex	4	100	15	71	19	76
5	Difficulties speaking	2	50	16	76	18	72
6	Ability to swallow the saliva on instruction	2	50	16	76	18	72
7	Ability to pass a water swallow test (without coughing)	3	75	15	71	18	72
8	Ability to perform mouth movements (e.g. tongue and lip movement)	3	75	13	62	16	64
9	Ability to cough reflexively or spontaneously	4	100	9	43	13	52
10	Ability to cough on instruction	2	50	7	33	9	36
11	Sound of the voice, for example, hoarse, aphonic (no voice)	2	50	5	24	7	28
12	Appetite	0	0	7	33	7	28
13	Weight changes	0	0	6	29	6	24
14	Oxygen desaturation	0	0	5	24	5	20
15	Other, please specify	0	0	2	10	2	8

Most participants (19 of 22, 86%) did not use a specific formal screening tool when identifying dysphagia. The use of two formal screening tools was, however, reported: ‘A water swallow test’ and an item referred to as ‘Post-operative Anterior Cervical Fusion Checklist B for swelling, dysphagia and dyspnoea’. Data regarding the timing of screening were not obtained.

### Dysphagia management

The intervention methods were divided into five subcategories, as presented in Table 3: enteral feeding, parenteral feeding, compensations, rehabilitation and referral.

**TABLE 3:** Dysphagia intervention.

Interventions	Items	Doctors (*n* = 4)		Registered nurses (*n* = 21)		Total (*n* = 25)	
		Number	%	Number	%	Number	%
Enteral feeds	Recommend a nasogastric (NG) tube	3	75	19	90	22	88
	Recommend a percutaneous endoscopic gastroscopy (PEG)	4	100	12	57	16	64
	Recommend a percutaneous endoscopic jejunostomy (PEJ)	0	0	1	5	1	4
Parenteral feeds	Recommend total parenteral nutrition(TPN)	0	0	13	62	13	52
	Recommend partial parenteral nutrition (PPN)	1	25	4	19	5	20
Compensations	Elevate the head of the bed to a 30° – 45° incline to prevent aspiration of secretions	4	100	20	95	24	96
	Ensure adequate fluid intake by putting up an intravenous drip	3	75	18	86	21	84
	Recommend positioning adjustments, for example, the patient must be seated upright during meals	4	100	15	71	19	76
	Improve oral hygiene	2	50	16	76	18	72
	Recommend a different (dysphagia) diet, for example, soft food, sloppy or puree food	3	75	15	71	18	72
	Maintain the patient upright for at least half an hour after a meal to prevent aspiration of refluxed material	2	50	15	71	17	68
	Recommend eating strategies, for example, taking small bites or sips	1	25	16	76	17	68
	Use a commercial thickener to thicken liquids or foods, for example, Nutilis/Thick & Easy	3	75	13	62	16	64
	Use a syringe to give the patient food or liquid orally	1	25	11	52	12	48
	Recommend head posture adjustments, for example, chin tuck, head rotation	0	0	7	33	7	28
	Other, please specify	0	0	2	10	2	8
Rehabilitation	Recommend exercises to strengthen the muscles of the swallowing mechanism, for example, tongue exercises	1	25	13	62	14	56
	Recommend swallow manoeuvres for functional training of the swallow, for example, Mendelsohn, effortful swallow	1	25	12	57	13	52
Referral	To a speech-language therapist	4	100	19	90	23	92
	To a clinical dietitian	3	75	11	52	14	56
	To an occupational therapist	1	25	13	62	14	56
	To a doctor	0	0	10	48	10	40
	To a physiotherapist	1	25	4	19	5	20
	Other healthcare professionals, please specify	1	25	1	5	2	8
	To a nurse	0	0	1	5	1	4

Intervention was predominantly provided through compensations, such as elevating the head of the bed, and referral to an SLT. Aside from this, almost half of the participants reported using syringe feeding as an alternative and just over half of the participants reported recommending total parenteral nutrition (TPN).

## Discussion

The results are discussed in relation to other research, where appropriate, and presented according to the study objectives, namely, dysphagia identification and management.

### Dysphagia identification

Dysphagia screening should be valid, reliable and repeatable, and therefore, the use of a formal screening test has been recommended as best practice in the United Kingdom, the United States and Canada Stroke Guidelines (Donovan et al., 2013; Hebert et al., 2016; Intercollegiate Stroke Working Party, 2016; Winstein et al., 2016). In the current study, most of the doctors and registered nurses did not use a formal screening test; this may be because of a lack of awareness of the available screening tests and their benefits, as none have been mentioned in the South African Stroke Guidelines (Bryer et al., 2010; Kiyani & Butt, 2014; Mubeen & Butt, 2014). The available formal screening tests for doctors and registered nurses were, however, all developed internationally, which may be why they are not mentioned in the South African Stroke Guidelines (Antonios et al., 2010; Bryer et al., 2010; Martino et al., 2009; Ostrofsky & Seedat, 2016). Doctors and registered nurses who are aware of these tests may also find that they are not suitable to the context in which they work and may choose not to use them (Ostrofsky & Seedat, 2016).

As an alternative to formal screening tests, the South African Stroke Guidelines indicate that wet voice, abnormal voluntary cough, abnormal phonatory quality, reduced level of consciousness and reduced laryngeal elevation or swallow should be screened before a formal swallowing assessment is performed (Bryer et al., 2010). The participants in the current study used various appropriate informal screening methods and indicators such as the presence of drooling, level of alertness and the ability to swallow saliva reflexively or spontaneously to determine the presence of dysphagia (Baroni et al., 2012; Horiguchi & Suzuki, 2011; Smith Hammond & Goldstein, 2006).

Important signs that the participants infrequently screened for were the sound of a patient’s voice and their ability to cough voluntarily. Vocal cord paralysis, which could occur in stroke patients, results in poor closure of the glottis and leads to dysphonia, reduced protection of the airway and therefore a higher risk for aspiration (Bhattacharyya, Kotz, & Shapiro, 2002). It is thus important to screen the sound of a patient’s voice to determine what their glottal closure and airway protection is like and to establish a baseline for comparison after the swallow. Changes in vocal quality may not be directly related to aspiration; however, the observation of a clear voice after the swallow indicates that aspiration is likely absent (Waito, Bailey, Molfenter, Zoratto, & Steele, 2011).

Coughing and swallowing share neuroanatomical substrates and thus an impaired voluntary cough after a stroke may also indicate impaired swallowing (Watts, Tabor, & Plowman, 2016). A weak voluntary cough indicates poor protection of the airway and reduced ability to expel aspirated material and has been correlated with dysphagia and aspiration (Smith Hammond & Goldstein, 2006; Watts et al., 2016; Ward et al., 2010). Therefore, it is important to screen a patient’s ability to cough voluntarily as an indication of the neuroanatomical integrity of the swallow and competence of airway protection.

In contrast to the above-mentioned important signs that were infrequently screened, the majority of the participants relied on the gag reflex as a determinant of the condition. As a single clinical feature, it has low sensitivity and specificity for identifying dysphagia and does not correlate to the results of a water or paste swallowing test (Bours, Speyer, Lemmers, Limburg, & De Wit, 2009; Henrique Dias Marques, Lúcia Zuma De Rosso, & André, 2008). Nakajima et al. (2010) found that 31% of the stroke patients in their non-oral group had an intact gag reflex. On the contrary, many healthy subjects may have an absent or suppressed gag reflex (Lim, Hew, Lau, Lim, & Tan, 2009). Doctors and registered nurses therefore should not over-rely on the gag reflex as a key item when identifying dysphagia.

The participants’ informal screening methods are possibly lacking because of insufficient knowledge of dysphagia and may lead to under-identification of dysphagia indicators. First-line contact medical practitioners would therefore benefit from using formal screening tools that are supported by evidence to guide their practice to accurately identify patients with dysphagia (Gandolfi et al., 2014).

### Dysphagia management

#### Enteral feeds

Both the doctors and registered nurses recommended enteral feeding via nasogastric (NG) tube as well as percutaneous endoscopic gastroscopy (PEG) tube. Most of the participants, however, recommended NG tube feeding. This is supported by Kenny and Singh (2015), who reported that PEG tubes should not be placed too soon after a stroke, as it poses a risk for mortality because of the underlying condition of a stroke. Nasogastric tubes are thus better for short-term enteral feeding, although it should not be *in situ* for longer than 4–8 weeks, as it could cause complications of its own such as nasal ulceration, increased reflux and increased risk of aspiration pneumonia if misplaced (Kenny & Singh, 2015). If patients cannot safely return to oral intake after a few weeks, PEG placement should be considered, as it provides better long-term nutritional support (Bouziana & Tziomalos, 2011).

#### Parenteral feeds

Total parenteral feeding (TPN) was recommended by more than half of the participants. While meeting nutritional goals, TPN can be potentially harmful, as it is associated with hepatic injury, thrombosis, hyperglycaemia and higher infection rates (Via & Mechanick, 2013). It should therefore only be advised in patients with severe malnutrition and concomitant gastrointestinal tract impairment (Via & Mechanick, 2013).

#### Compensations

The participants made use of various compensation methods that were in line with the literature (Table 3) (Bryer et al., 2010; Hines et al., 2011; Metheny, Davis-Jackson, & Stewart, 2010). However, the participants also indicated the use of the potentially dangerous practice of syringe feeding. Although there has been limited research on the topic, syringe feeding is not recommended, nor appropriate, for stroke patients with dysphagia (Lazarus & Kulpa, 1996). Syringe feeding could lead to premature spillage of the bolus into the pharynx and subsequent aspiration, if the bolus size is not carefully controlled and strategically placed on the floor of the mouth (Lazarus & Kulpa, 1996; Wakasugi et al., 2008). The fact that almost half of the participants use this as a management method indicates that they are not aware of the potential risks associated with syringe feeding.

#### Rehabilitation

Rehabilitation is aimed at improving the function of the swallowing mechanism to advance recovery (Rogus-Pulia & Robbins, 2013; Vose, Nonnenmacher, Singer, & González-Fernández, 2014). In the current study, more than half of the participants indicated that they would recommend rehabilitation exercises such as muscle strengthening exercises (e.g. for the tongue) and swallowing manoeuvres (for functional training, e.g. Mendelsohn). Researchers have indicated that other healthcare professionals such as registered nurses could perform rehabilitation exercises after a full swallowing assessment has been performed by an SLT, if they have had the necessary specialised training to do so (Hines et al., 2011; Perry, Hamilton, Williams, & Jones, 2013). Doctors and registered nurses should, however, caution against prescribing therapeutic interventions such as rehabilitation exercises, as this does not fall within their scope of practice but is solely the SLT’s role (ASHA, 2015; Perry et al., 2013).

#### Referral

Dysphagia is best managed in a multidisciplinary team; therefore, appropriate and timely referral is very important (Gandolfi et al., 2014). As dysphagia could lead to malnutrition, it is essential for a dietitian to manage nutritional intake (Blackwell & Littlejohns, 2010). Occupational therapists also provide valuable interventions like the adaptation of feeding utensils and exercises to improve self-feeding, which may reduce the risk of pneumonia (Bryer et al., 2010; Langmore, Skarupski, Park, & Fries, 2002; Rogus-Pulia & Robbins, 2013). Most of the participants referred patients with dysphagia to an SLT. Participants appear not to be aware of the role of other members of the multidisciplinary team, specifically occupational therapists and dietitians as they inadequately referred to them.

In summary, the doctors and registered nurses used various appropriate management methods such as elevating the head of the bed, referring to an SLT, recommending an NG tube or positioning adjustments and placing an intravenous drip (Bryer et al., 2010; Carnaby et al., 2006; Hines et al., 2011; Kenny & Singh, 2015; Metheny et al., 2010; Perry et al., 2013; Seedat & Penn, 2016). However, they also reported recommending rehabilitation exercises and manoeuvres, which is not within their scope of practice, and using potentially harmful methods such as parenteral and syringe feeding (Lazarus & Kulpa, 1996; Via & Mechanick, 2013; Wakasugi et al., 2008). This testifies that doctors and registered nurses may have insufficient knowledge of dysphagia to intervene optimally and may not be sure of their role and scope of practice within dysphagia management.

### Significance and limitations

Doctors and registered nurses identify and manage dysphagia to the best of their knowledge, but risk under-identifying patients or harming them, because their knowledge of dysphagia may be insufficient. If doctors and registered nurses are expected to share the task of dysphagia identification and management, they should receive tertiary and in-service training to improve their knowledge, which could improve their practice and reduce the use of dangerous and inappropriate practices for the safety of the patients. Dysphagia practices could also be improved through standardisation and the use of formal screening tools such as the South African dysphagia screening tool (SADS) (Ostrofsky & Seedat, 2016).

The data from this study are useful but with limited generalisability because of poor external validity as a result of non-probability sampling and a poor response rate. Notably, the data are of only a single hospital site. The survey design was furthermore limited to self-reported data of dysphagia identification and management practices, which could be influenced by social desirability bias and therefore may not reflect what is truly performed in practice (Grimm, 2010). It is acknowledged that there may be concerns regarding the wording of the tool and the use of the same questionnaire for registered nurses and doctors who have different scopes of practice.

The design of the tool was, however, informed by the pilot study conducted. We also recognise that the viability of the recommendation for doctors and registered nurses to conduct the initial dysphagia screening needs to be explored, as it may over-burden these professionals who face staff shortages of their own.

### Recommendations

The following recommendations for future research bear relevance:

1. Given the high prevalence (56%) of dysphagia in stroke patients in SA, this study should be repeated and comparisons drawn among private and public hospitals across SA (Blackwell & Littlejohns, 2010).
2. This study should also be conducted with a larger study population to enable random sampling, which will allow generalisation of the results and improve external validity.
3. Studies are needed to investigate the effect of: (1) in-service training; (2) the use of a formal screening tool; and (3) discontinuing inappropriate practices, on doctors and registered nurses’ dysphagia practices, as well as on pneumonia and mortality rates associated with dysphagia.
4. There is a need to develop a South African dysphagia screening tool (e.g. Ostrofsky and Seedat’s South African Dysphagia Screening, 2016), which can also be used by doctors and registered nurses.

## Summary and conclusion

Doctors and registered nurses mainly used informal screening methods to identify dysphagia, and neglected important indicators, such as voluntary cough and vocal quality, which could lead to the under-identification of dysphagia. Management methods such as parenteral nutrition and syringe feeding, which can potentially lead to higher infection rates and aspiration, respectively, were also used (Lazarus & Kulpa, 1996; Via & Mechanick, 2013; Wakasugi et al., 2008). The participants furthermore took it upon themselves to recommend rehabilitation exercises and manoeuvres, which is beyond their scope of practice (ASHA, 2015; Perry et al., 2013). Doctors and registered nurses could benefit from dysphagia training during and after their undergraduate programmes, including the use of formal screening tools. As primary practitioners in dysphagia management, SLTs will need to facilitate tertiary as well as in-service training for doctors and registered nurses, with a focus on their particular role within the multidisciplinary dysphagia management team. This may result in more timely and appropriate referrals and reduce the use of potentially harmful management practices, which, in turn, could reduce the occurrence of complications such as pneumonia and mortality.

## Appendix 1

Dysphagia Management Questionnaire

The management of hospitalised adults with neurogenic dysphagia: A descriptive study

Dysphagia refers to any swallowing difficulties and/or disorders. Dysphagia is a common complication after a stroke.

Instructions:

- Please complete the questionnaire **on your own** and **do not share** your answers with others
- Please complete **all** of the applicable questions
- **Please tick (✓) the appropriate box(es)**

### Section 1: Identification of dysphagia refers to the formal screening and/or informal observation of dysphagia signs and symptoms.

#### 1.1 Have you ever identified dysphagia in stroke patients?

□ Yes     □ No

***If no to question 1.1, please proceed to Section 3***

#### 1.2 Do you use a specific screening test or checklist to assess the presence or absence of dysphagia? (choose one answer)

□ Yes     □ No

If yes, please specify the name of the screening test or checklist_______________________

#### 1.3 What do you assess or observe (formally or informally) to determine whether or not the patient has dysphagia? (Please tick all the relevant boxes)

□ Level of alertness
□ Presence of a gag reflex
□ Sound of their voice, for example, hoarse, aphonic (no voice)
□ Difficulties speaking
□ Ability to perform mouth movements (e.g. tongue and lip movement)
□ Ability to cough reflexively or spontaneously
□ Ability to cough on instruction
□ Presence of drooling
□ Weight changes
□ Ability to swallow their saliva reflexively or spontaneously
□ Ability to swallow their saliva on instruction
□ Ability to pass a water swallow test (without coughing)
□ Appetite
□ Oxygen desaturation
□ Other, please specify ______________________

#### 1.4 Which of the following would you say are signs or symptoms of dysphagia? (Please tick all the relevant boxes)

□ Eating fast
□ Food or liquid falling out of the mouth or coming through the nose
□ Coughing or throat clearing before, during or after swallowing
□ Loss of appetite and/or reduced intake
□ Nausea and/or vomiting
□ Difficulty getting the food or medication down
□ Food left over in the mouth after swallowing
□ Voice changes after swallowing
□ Respiratory problems
□ Eating too much
□ Other, please specify ______________________

#### 1.5 What other factors may put stroke patients at an increased risk for dysphagia? (Please tick all the relevant boxes)

□ Reduced level of consciousness
□ Older than 60 years
□ Over weight
□ Lung disease or pneumonia
□ Comprehension difficulties
□ Needs oxygen
□ Stroke in the left hemisphere (right-sided weakness)
□ A brainstem stroke
□ A weak cough
□ Needs oral-pharyngeal suctioning
□ Slurred speech or difficulties speaking
□ Small mouth
□ Has had more than one stroke
□ Has another muscular or neurological disease, for example, Parkinson’s disease, Alzheimer’s disease, Dementia and so on.

### Section 2: Intervention in dysphagia refers to the techniques used to maintain or improve the safety of the patient’s current intake. The aim is to prevent complications, for example, aspiration pneumonia, dehydration or malnutrition.

#### 2.1 What do you do once you have identified that a patient has dysphagia? (Please tick all the relevant boxes)

**Enteral feeds**

□ Recommend a nasogastric (NG) tube
□ Recommend a percutaneous endoscopic gastroscopy (PEG)
□ Recommend orogastric feeding
□ Recommend a percutaneous endoscopic jejunostomy (PEJ)
□ Other, please specify ________________________

**Parenteral feeds**

□ Recommend partial parenteral nutrition (PPN)
□ Recommend total parenteral nutrition (TPN)
□ Other, please specify ________________________

**Compensations**

□ Use a commercial thickener to thicken liquids or foods, for example, Nutilis/Thick & Easy
□ Use a syringe to give the patient food or liquid orally
□ Ensure adequate fluid intake by putting up an intravenous drip
□ Elevate the head of the bed to a 30° – 45° incline to prevent aspiration of secretions
□ Improve oral hygiene
□ Recommend a different (dysphagia) diet, for example, soft food, sloppy or puree food
□ Recommend positioning adjustments, for example, the patient must be seated upright during meals
□ Maintain the patient upright for at least half an hour after a meal to prevent aspiration of refluxed material
□ Recommend head posture adjustments, for example, chin tuck, head rotation
□ Recommend eating strategies, for example, taking small bites or sips
□ Other, please specify ________________________

**Rehabilitation**

□ Recommend swallow manoeuvres for functional training of the swallow, for example, Mendelsohn, effortful swallow
□ Recommend exercises to strengthen the muscles of the swallowing mechanism, for example, tongue exercises
□ Other, please specify ________________________

**Referral**

□ To a doctor
□ To a nurse
□ To a clinical dietitian
□ To a physiotherapist
□ To an occupational therapist
□ To a speech therapist
□ Other healthcare professional, please specify___________________________

#### 2.2 When a patient is tube fed (e.g. PEG/NG) because of a swallowing difficulty, how do you decide if they can eat orally again? (Please tick all the relevant boxes)

□ Do a swallowing screening
□ Refer the patient to a speech therapist
□ Give the patient an oral feeding trial, for example, food or water
□ I do not do or decide this

### Section 3: Training and experience

#### 3.1 In which ward(s) have you worked?

□ ICU
□ High care
□ Cardiothoracic ward (E ward)
□ Medical ward (F ward)
□ All of the above

#### 3.2 Where did you receive your qualification?

□ College. Please specify_________________________________
□ University. Please specify_______________________________
□ Other. Please specify___________________________________

##### 3.2.1 Did you receive any information on dysphagia during your studies?

□ Yes     □ No

##### 3.2.2 If yes to question 3.2.1, do you feel that the information was sufficient?

□ Yes     □ No

#### 3.3 Did you learn about dysphagia anywhere else?

□ Yes     □ No

##### 3.3.1 If yes to question 3.3, where did you learn about dysphagia? (Please tick all the relevant boxes)

**Informal method**

□ Peer, for example, colleague
□ Mentor or manager, for example, unit manager or clinical lead
□ Self-study, for example, journal article or book
□ Other, please specify___________________________

**Formal method**

□ Continuing professional development (CPD) activity, please specify ____________
□ Other, please specify___________________________

#### 3.4 How many years of work experience do you have?

□ <5 years
□ 5–9 years
□ 10–19 years
□ 20+ years
